# Unveiling the oncogenic role of LZTS1 in colorectal cancer

**DOI:** 10.1111/jcmm.18441

**Published:** 2024-07-18

**Authors:** Yuanchun Xu, Daniele Pepe, Shu Yao, Loubna Boudhan, Sara Verbandt, Ting Pu, John W. M. Creemers, Maoxuan Liu, Sabine Tejpar, Zongsheng He, Jingjing Zhu, Yaling Wang

**Affiliations:** ^1^ Department of Neurosurgery Daping Hospital, Army Medical University Chongqing China; ^2^ Department of Nursing Daping Hospital, Army Medical University Chongqing China; ^3^ Laboratory for Disease Mechanisms in Cancer KU Leuven Leuven Belgium; ^4^ Department of Gastroenterology Daping Hospital, Army Medical University Chongqing China; ^5^ Ludwig Institute for Cancer Research Brussels Belgium; ^6^ de Duve Institute, UCLouvain Brussels Belgium; ^7^ Walloon Excellence in Life Sciences and Biotechnology Brussels Belgium; ^8^ Digestive Oncology KU Leuven Leuven Belgium; ^9^ Department of Human Genetics KU Leuven Leuven Belgium; ^10^ Center for Protein and Cell‐Based Drugs, Institute of Biomedicine and Biotechnology, Shenzhen Institute of Advanced Technology, Chinese Academy of Sciences Shenzhen China

**Keywords:** AKT, colorectal cancer, EMT, LZTS1

## Abstract

Although leucine zipper tumour suppressor 1 (LZTS1) has been considered a potential tumour suppressor, accumulating evidence suggests that LZTS1 is highly expressed in many cancer types. To unravel the exact role of LZTS1 in colorectal carcinogenesis, we performed the bioinformatic analysis of LZTS1, including expression differences, correlations between expression levels and survival, methylation status of LZTS1 promoter and related cellular pathways based on TCGA dataset, GEO databases and our own CRC patient cohort. Furthermore, we confirmed the oncogenic function of LZTS1 in human mammalian cells by employing a series of assays including tissue microarray, immunoblotting, cell proliferation and migration assay. We found that the expression of LZTS1 is higher in tumour samples compared to paired normal tissue in CRC cancer and its different clinical subtypes, which is, at least in part, due to the low methylation status of LZTS1 promoter in CRC tumour samples. Functional analysis identified the close relationship between high expression of LZTS1 and PI3K‐AKT pathway and the epithelial–mesenchymal transition (EMT) process. Consistently, we found that the expression of LZTS1 positively correlated with the expression PIK3CD, N‐cadherin in CRC tumour samples, while the expression of LZTS1 negatively correlated with the expression of E‐cadherin and PTEN in CRC tumour samples. Experimental data further confirmed that overexpression of LZTS1 upregulated activity of AKT and promoted EMT process. Furthermore, depletion of LZTS1 repressed the proliferation and migration rate of CRC cells. Thus, this study indicates that LZTS1 plays an oncogenic role in colorectal carcinogenesis.

## 1 BACKGROUND

Colorectal cancer (CRC) is one of the most common types of cancer worldwide with high morbidity and mortality.^1^ Although the increasing understanding of CRC pathophysiology has expanded the treatment options to include radiotherapy, chemotherapy, immunotherapy and targeted therapy, CRC remains a serious threat to life for millions of people globally.^2^ Therefore, it is urgent to unravel the aetiology of CRC, which will contribute to identifying novel therapeutic targets.

At molecular level, colorectal carcinogenesis arises from abnormal expression of oncogenes and tumour suppressors, which mostly results from genetic mutations and epigenetic alterations.^3^ The mutations of genes like TP53, for instance, have been found in more than half of CRC tumour samples.^4^ Accumulating evidence suggests that the abnormal epigenetic modulations of these driver genes are demonstrated as important regulators for CRC.^5^ Thus, these potential regulators could be potential targets for CRC treatment. Intriguingly, some genes could assume the oncogenic or tumour suppressive role in different stages of cancer or different subtypes of cancer. For instance, TGFb1 functions as a tumour suppressor by suppressing cell growth and inducing apoptosis at the early stage of cancer, while it acts as an oncogene by inducing EMT and regulatory T‐cell proliferation at the late stage of CRC.^6^, ^7^ He et al. also reported that Furin, a proprotein convertase, serves a double role in CRC.^8^, ^9^ These studies suggest that more work is still needed to clarify the distinct roles of genes in cancer, providing precise guidance for cancer treatment.

The leucine zipper tumour suppressor (LZTS) family comprises three members: LZTS1, LZTS2 and LZTS3. Several studies show that the expression of LZTS members seems to be downregulated in tumour samples of breast cancer and lung cancer,^10^, ^11^ indicating that LZTS has a tumour suppressive function in these cancers. Furthermore, deletion of LZTS1 in mouse embryos predisposes mice to cancer development by accelerating mitotic progression.^12^ In human cancer cell lines, repression of LZTS1 causes aggressive phenotypes by regulating the PI3K‐AKT pathway.^13^, ^14^ On the other hand, emerging evidence suggests that LZTS1 shows higher expression in pancreatic tumour tissue compared to paired normal tissue, indicating the oncogenic function of LZTS1 in human cancers.^15^ Furthermore, inhibition of LZTS1 reduced the activity of AKT and its downstream target glycogen synthase kinase 3 β (GSK‐3β) in pancreatic cancer cells, while overexpressed LZTS1 led to upregulated activity of AKT and GSK‐3β. These studies suggest that the role of LZTS1 could vary from cancer to cancer.

Here, our results show that the expression of LZTS1 is higher in CRC tumour samples compared to their paired normal tissue in both public datasets and our cohort. Accordingly, CRC tumour samples harbour a higher promoter methylation level of LZTS1 than that in the paired normal tissue. Bioinformatic analysis and in vivo assay show that LZTS1 contributes to tumorigenesis by increasing the activity of AKT and EMT. Furthermore, depletion of LZTS1 repressed the proliferation and migration rate of CRC cells. Our study suggests that LZTS1 functions as an oncogene in CRC.

## 2 RESULTS

### 2.1 The increased expression of LZTS1 in CRC and other cancer types

**FIGURE 1:**
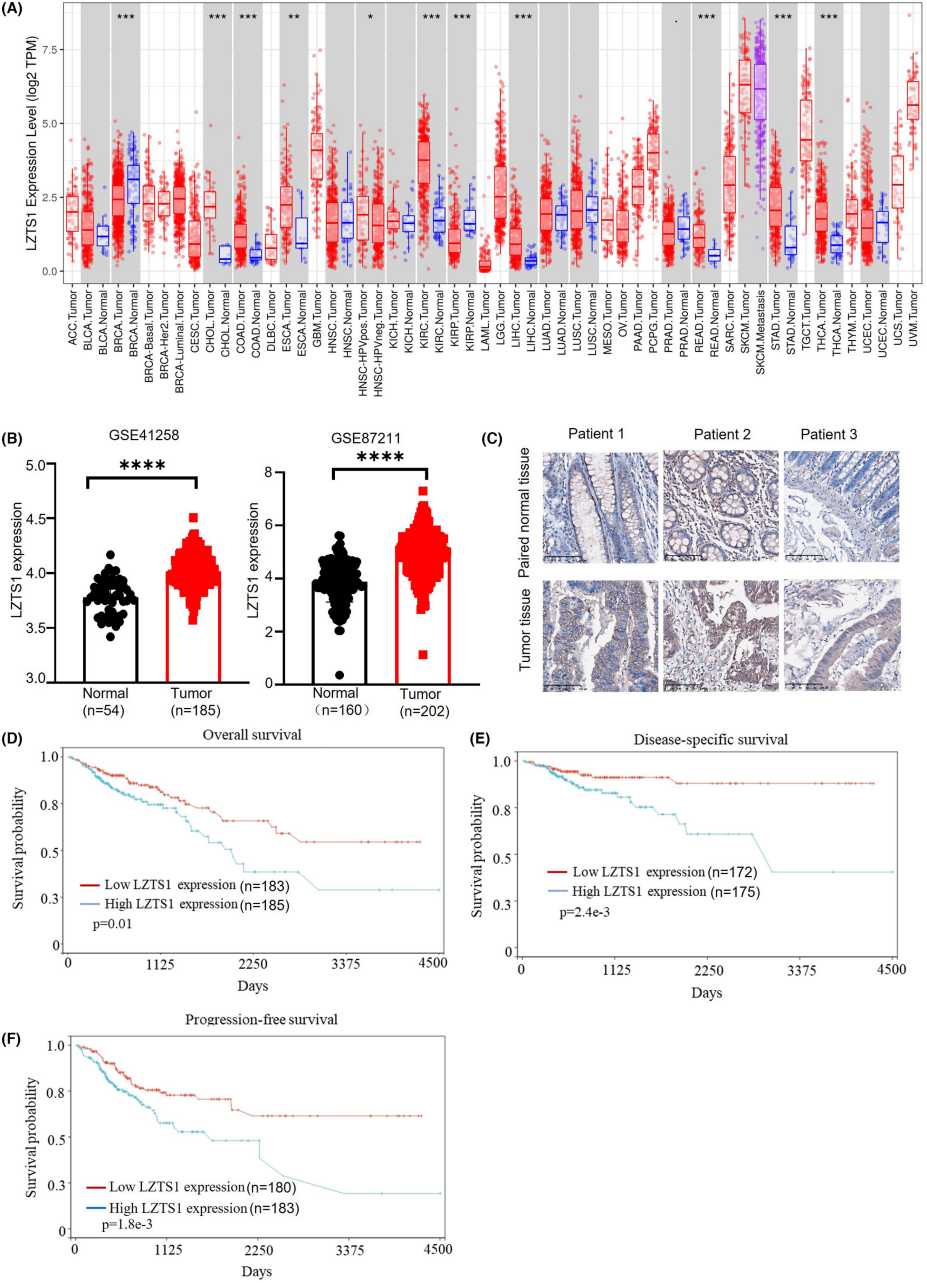
LZTS1 is upregulated in CRC. (A) Transcriptional levels of LZTS1 in different tumour types from the TCGA database summarized by TIMER database. (B) Box plot of LZTS1 expression in two independent GEO datasets (GSE41258 and GSE87211). (C) The protein levels of LZTS1 were checked by immunohistochemistry in tumour and paired normal tissue of CRC patients. Scale bar: 100μm. (D) Overall survival (OS) analysis of CRC patients from the TCGA database stratified by LZTS1 expression using the K–M plotter tool. (E) Disease‐specific survival (DSS) analysis of CRC patients from the TCGA database grouped by LZTS1 expression using the Kaplan–Meier plotter tool. (F) Progression‐free survival (PFS) analysis of CRC patients from the TCGA database grouped by LZTS1 expression using the K–M plotter tool. A two‐tailed Student's *t*‐test was performed for (B) log‐rank test was performed for (D–F). *p < 0.05, **p < 0.01, ***p < 0.001, ****p < 0.0001.

Since the expression of LZTS1 has been reported as a potential tumour suppressor gene in several cancer types, we first analysed the expression of LZTS1 in pan‐cancer based on the TCGA database. Our analysis showed that the expression of LZTS1 was significantly upregulated in eight types of cancer including COAD and READ (Figure 1A). On the other hand, the BRCA and KIRP tumour samples displayed downregulated LZTS1 expression compared to normal tissues (Figure 1A). Furthermore, we found that the expression of LZTS1 was significantly increased in CRC tumour samples based on two individual GEO datasets (Figure 1B). To confirm the expression of LZTS1 in CRC tissue at the protein level, we checked its expression in three paired CRC patients' samples by means of immunohistochemistry. As Figure 1C shows, LZTS1 was highly expressed in CRC tumour samples compared to paired adjacent normal tissue in these three paired CRC samples, indicating that LZTS1 expression is highly upregulated in CRC tumour samples.

Next, we analysed the relationship between LZTS1 expression and CRC patient' prognosis. We observed that high LZTS1 expression was correlated with poor overall survival (OS) in 368 CRC samples (Figure 1D). Furthermore, LZTS1 overexpression was also associated with worse disease‐specific survival (DSS) (Figure 1E) and a progression‐free interval (Figure 1F) among these patients. These data reveal that upregulated LZTS1 expression is linked to tumour progression in CRC.

### 2.2 Expression of LZTS1 in different CRC subgroups

**FIGURE 2:**
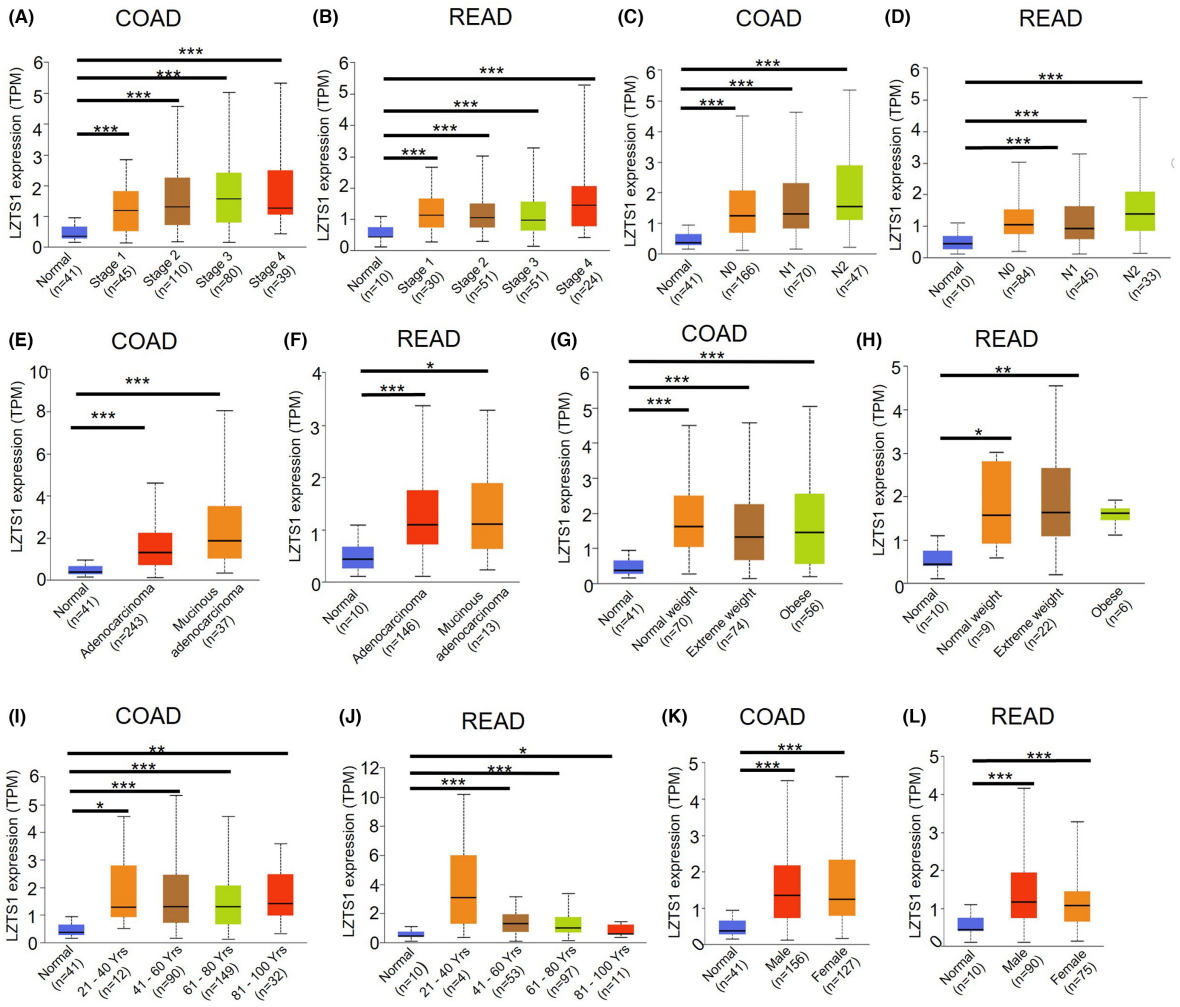
The correlation between LZTS1 expression and distinct clinicopathological status in TCGA COAD and READ dataset. (A, B) The correlation between LZTS1 expression and tumour stage in TCGA COAD (A) and READ (B) dataset. (C, D). The correlation between LZTS1 expression and lymph nodes metastasis in TCGA COAD (C) and READ (D) dataset. (E, F). The correlation between LZTS1 expression and cancer subtype in TCGA COAD (E) and READ (F) dataset. (G, H). The correlation between LZTS1 expression and patients' body weight in TCGA COAD (G) and READ (H) dataset. (I, J). The correlation between LZTS1 expression and patients' age in TCGA COAD (I) and READ (J) dataset. (K, L). The correlation between LZTS1 expression and patients' gender in TCGA COAD (K) and READ (L). The Welch's T‐test was used to estimate the significance of differences in expression levels between normal and primary tumours or tumour subgroups based on clinicopathological features. *p < 0.05, **p < 0.01, ***p < 0.001.

Next, we evaluated the correlation between LZTS1 expression and clinical features of other CRCs (colon adenocarcinoma: COAD; rectal adenocarcinoma: READ). In both COAD and READ, LZTS1 expression revealed a substantial upregulation trend as tumour grade increased, compared to normal tissues (Figure 2A,B). There is also the case in the N stage of COAD and READ, as LZTS1 expression showed a strong up‐regulation trend as the N stage advanced (Figure 2C,D). As for adenocarcinoma and mucus adenocarcinoma, both COAD and READ showed enhanced LZTS1 expression compared to normal tissue, along with increased trend of LZTS1 in mucus adenocarcinoma (Figure 2E,F). Compared to normal tissue, we observed that LZTS1 expression was significantly higher in COAD patients despite their body weight (Figure 2G). Similarly, LZTS1 expression showed the upregulation trend in READ tumours, although there was no significant difference between normal and obese subgroups in terms of LZTS1 expression (Figure 2H). As Figure 2I displayed, age is an important factor for LZTS1 expression in patients with COAD or READ, but the tendency was the opposite. For the COAD patients, LZTS1 expression was high across all age groups, with the highest among 81–100 years subgroup (Figure 2I), while LZTS1 expression showed a downregulation trend among READ patients as the age increased (Figure 2J). In both male and female patients with COAD or READ, LZTS1 expression was upregulated in tumorous tissues compared to normal surrounding tissues (Figure 2K,L). We also observed that LZTS1 expression was higher in male than female. The results reveal that LZTS1 is potentially related to the advancement of CRC.

### 2.3 Methylation landscape of LZTS1 in CRC

**FIGURE 3:**
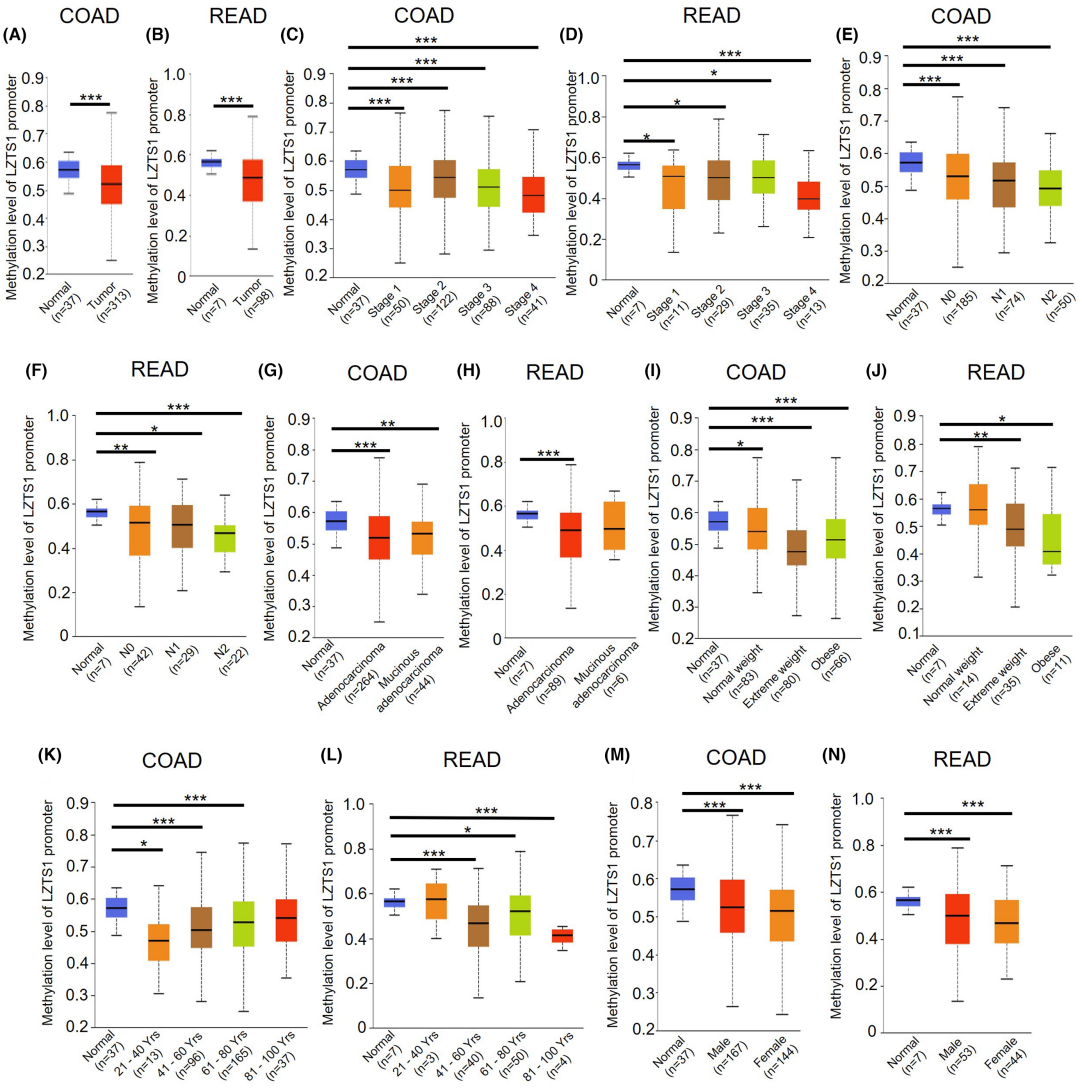
The methylation pattern of LZTS1 promoter in CRC samples from TCGA database. (A, B). The methylation level of LZTS1 promoter in TCGA COAD (A) and READ (B) dataset. (C, D). The methylation level of LZTS1 promoter in tumour sample with different stage from TCGA COAD (C) and READ (D) dataset. (E, F). The methylation level of LZTS1 promoter in tumour sample with different lymph nodes metastasis status from TCGA COAD (E) and READ (F) dataset. (G, H). The methylation level of LZTS1 promoter in different cancer subtype from TCGA COAD (G) and READ (H) dataset. (I, J). The methylation level of LZTS1 promoter in tumour sample with different patients' body weight in TCGA COAD (I) and READ (J) dataset. (K, L). The methylation level of LZTS1 promoter in tumour sample with different patients' age in TCGA COAD (K) and READ (L) dataset. (M, N). The methylation level of LZTS1 promoter in tumour sample from male patients or female patients in TCGA COAD (M) and READ (N) dataset. The Welch's T‐test was used to measure the significance of hypo‐/hyper‐methylation status of promoter DNA between normal and primary tumours or tumour subgroups based on clinicopathological features. *p < 0.05, **p < 0.01, ***p < 0.001.

DNA methylation is an essential epigenetic mechanism for regulating gene expression, and a high DNA methylation level often suppresses transcriptional activation of genic regions.^16^ To understand whether the methylation pattern of LZTS1 caused the different expression levels of LZTS1 in COAD and READ as well as paired normal tissue, we analysed the methylation levels of LZTS1 promoter in COAD and READ. As Figure 3A,B show, the methylation level of LZTS1 promoter was lower in COAD and READ tumour samples compared to normal surrounding tissue, consistent with the finding that higher expression of LZTS1 was observed in COAD and READ tumour samples than in those of normal tissue.

Then, we further analysed the methylation pattern of LZTS1 in distinct subgroups of COAD and READ. In keeping with the expression pattern of LZTS1 in COAD and READ, the methylation level of the LZTS1 promoter was significantly lower in tumour samples of COAD or READ with different tumour grades or N stage, compared to normal tissues (Figure 3C–F). Furthermore, the tumour samples at an advanced stages showed the lowest methylation level of LZTS1. As for adenocarcinoma and mucus adenocarcinoma, both COAD and READ showed decreased LZTS1 expression compared to normal tissue (Figure 3G,H). Additionally, diverse body weight, ages and genders of COAD or READ all patients had a lower LZTS1 gene promoter methylation level than the normal group (Figure 3I–N). Altogether, decreased methylation levels of LZTS1 promoter could result in the increased expression of LZTS1 in CRC.

### 2.4 The expression of LZTS1 in Chinese CRC cohort

**FIGURE 4:**
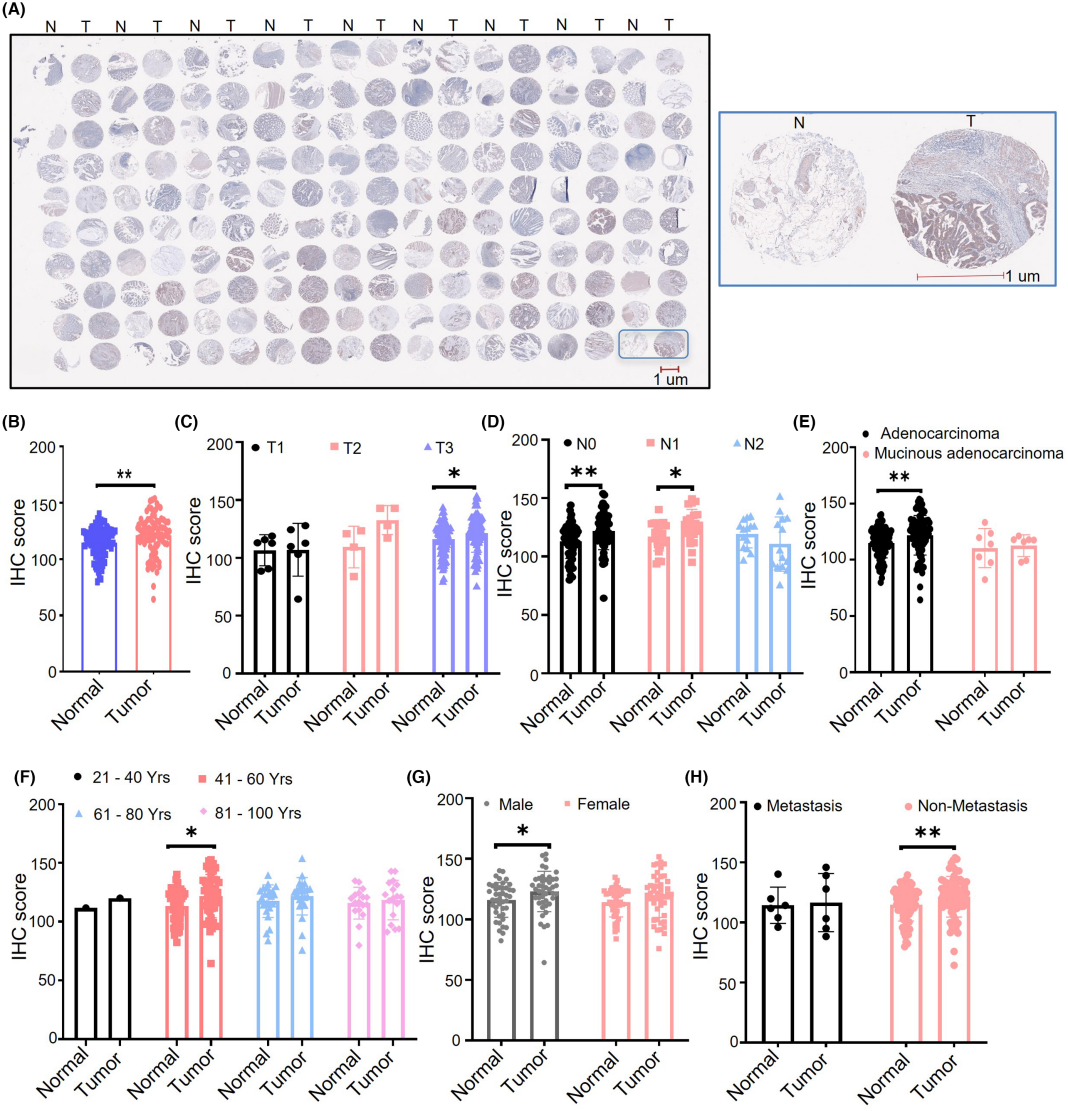
The protein level of LZTS1 in individual CRC tumour samples cohort. (A) The immunohistochemistry of LZTS1 in 180 tissue spots from 90 CRC patients (left panel). Representative images of LZTS1 immunohistochemistry in tumour tissue (T) and paired normal tissue (N) (right panel). (B) The quantification of LZTS1 immunohistochemistry in tumour tissue and paired normal tissue based on the immunohistochemistry score. (C) The LZTS1 expression in CRC tumour samples with different stage. (D) The LZTS1 expression in CRC tumour samples with different extent of lymph nodes metastasis. (E) The LZTS1 expression in different CRC subtype. (F) The LZTS1 expression in tumour sample with different patients' age. (G) The LZTS1 expression in tumour sample from male patients or female patients. (H) The LZTS1 expression in tumour sample with or without distant metastasis. A two‐tailed Student's *t*‐test was performed for (B–H) *p < 0.05, **p < 0.01.

To confirm the bioinformatic analysis of LZTS1 expression in CRC, we examined the expression of LZTS1 in our own CRC cohort containing of 90 CRC patients. Based on the IHC staining of LZTS1 in a tissue microarray with 90 cases of CRC and paired adjacent colorectal tissues (Figure 4A), we found that the protein expression of LZTS1 was significantly upregulated (*p* < 0.01) in CRC tissues compared to adjacent colorectal tissues (Figure 4B), consistent with the high expression of LZTS1 in CRC tissues from TCGA and GEO datasets. Next, we analysed the expression of LZTS1 in different CRC subgroups. Across different tumour grades (T1–T3), increased expression of LZTS1 was observed in CRC tissues compared to normal tissues (Figure 4C). Interestingly, different N stages showed different trends in terms of LZTS1 expression. In the N0 and N1 stages, LZTS1 expression was significantly increased in CRC compared to the adjacent colorectal tissues, while its expression showed a decreased trend in the N2 stage (Figure 4D). Although both adenocarcinoma tissues and mucinous adenocarcinoma tissues showed higher LZTS1 expression than that of the paired normal tissues, the increased degree of LZTS1 expression in adenocarcinoma tissues was much stronger than mucinous adenocarcinoma tissues (Figure 4E). As Figure 4F displayed, LZTS1 expression had an upregulated trend in CRC tissues compared to the normal tissues in distinct age subgroups (Figure 4F). In male CRC patients, LZTS1 expression was significantly increased in tumour tissues versus normal tissues, and its expression level also showed an upregulation trend in female CRC patients (Figure 4G). In the CRC patients with distant metastasis, despite the limited sample size, we still observed a slight upregulation trend in the tumour tissues compared to normal tissues (Figure 4H). Furthermore, in the CRC patients without metastasis, LZTS1 expression was significantly upregulated in the tumour tissues compared to the normal tissues (Figure 4H). These results further support the hypothesis that LZTS1 contributes to colorectal carcinogenesis in our cohort.

### 2.5 Upregulated LZTS1 expression enhances the activity of PI3K‐AKT and EMT pathways in CRC

**FIGURE 5:**
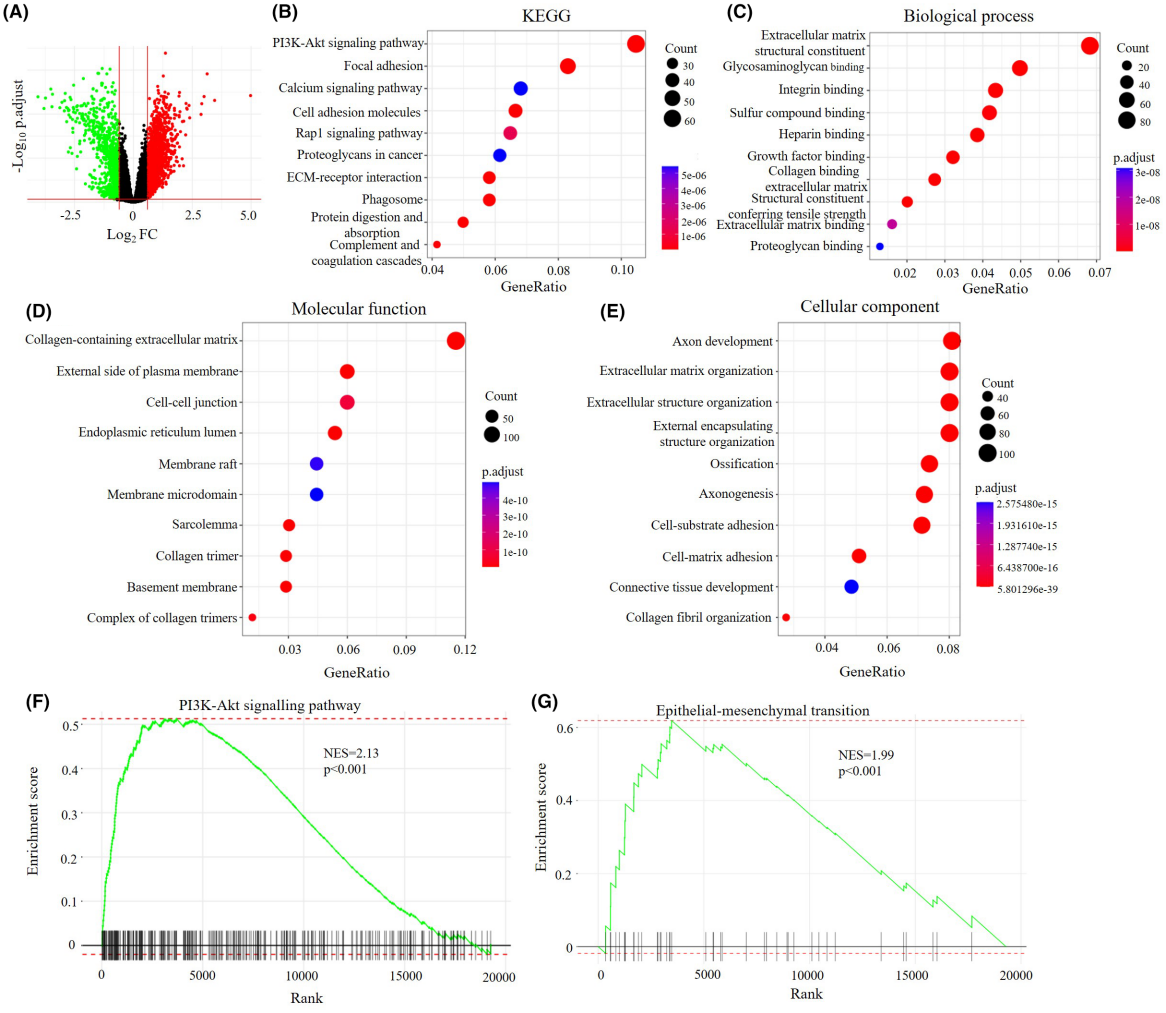
LZTS1 is vital in PI3K‐AKT pathway and EMT process. (A) Volcano plot indicates DEGs by comparing LZTS1^low^ and LZTS1^high^ groups from the TCGA COAD+READ datasets. Green dot: downregulated DEGs; red dot: upregulated DEGs. (B) Pathways enrichment of 1635 upregulated DEGs by KEGG analysis. (C–E). Biological process, molecular function and cellular component of 1635 upregulated DEGs by GO analysis. (F) GSEA shows that processes of PI3K‐AKT singling pathway (F) and epithelial‐mesenchymal transition (EMT) (G) are significantly enriched in patients with high LZTS1 expression.

We next aimed to unravel the biological function of LZTS1 in CRC. To do so, we compared differentially expressed genes (DEGs) in the TCGA COAD‐READ cohort between the LZTS1^high^ and LZTS1^low^ groups. Among these DEGs, we identified 1635 upregulated DEGs and 1622 downregulated DEGs (Figure 5A; Table S1). KEGG pathway enrichment analysis showed that the PI3K‐AKT signalling pathway, focal adhesion and other signalling pathways were significantly enriched in the upregulated DEGs (Figure 5B). In contrast, Alzheimer disease, prion diseases and other pathways were significantly enriched in the downregulated DEGs (Figure S1A). The GO analysis revealed that the biological processes associated with these upregulated DEGs were extracellular matrix structural constituent, integrin binding and other biological processes (Figure 5C), which were related to epithelial–mesenchymal transition (EMT). Furthermore, GO molecular function analysis identified that these upregulated DEGs were enriched in EMT‐related molecular functions including collagen‐containing extracellular matrix and cell–cell junction (Figure 5D). These upregulated DEGs were closely related to extracellular matrix organization and extracellular structure organization, both of which played important roles in EMT (Figure 5E), In contrast, these downregulated DEGs were enriched into distinct biological processes, molecular functions and cellular components (Figure S1B–D). These data suggest that LZTS1 contributes to PI3K‐AKT signalling pathways activation and EMT biological processes.

We then analysed the potential pathways associated with the LZTS1 expression level via GESA analysis. Consistent with KEGG analysis, LZTS1 expression was positively associated with the activity of the PI3K‐AKT signalling pathway (Figure 5F), indicating that LZTS1 could upregulate the activity of the PI3K‐AKT signalling pathway. Furthermore, we observed that LZTS1 expression was positively associated with the EMT pathway (Figure 5G), in keeping with GO analysis. This evidence reveals that LZTS1 plays a critical role in PI3K‐AKT and EMT signalling pathways.

### 2.6 LZTS1 overexpression strengthens the activity of the PI3K‐AKT pathway and EMT process

**FIGURE 6:**
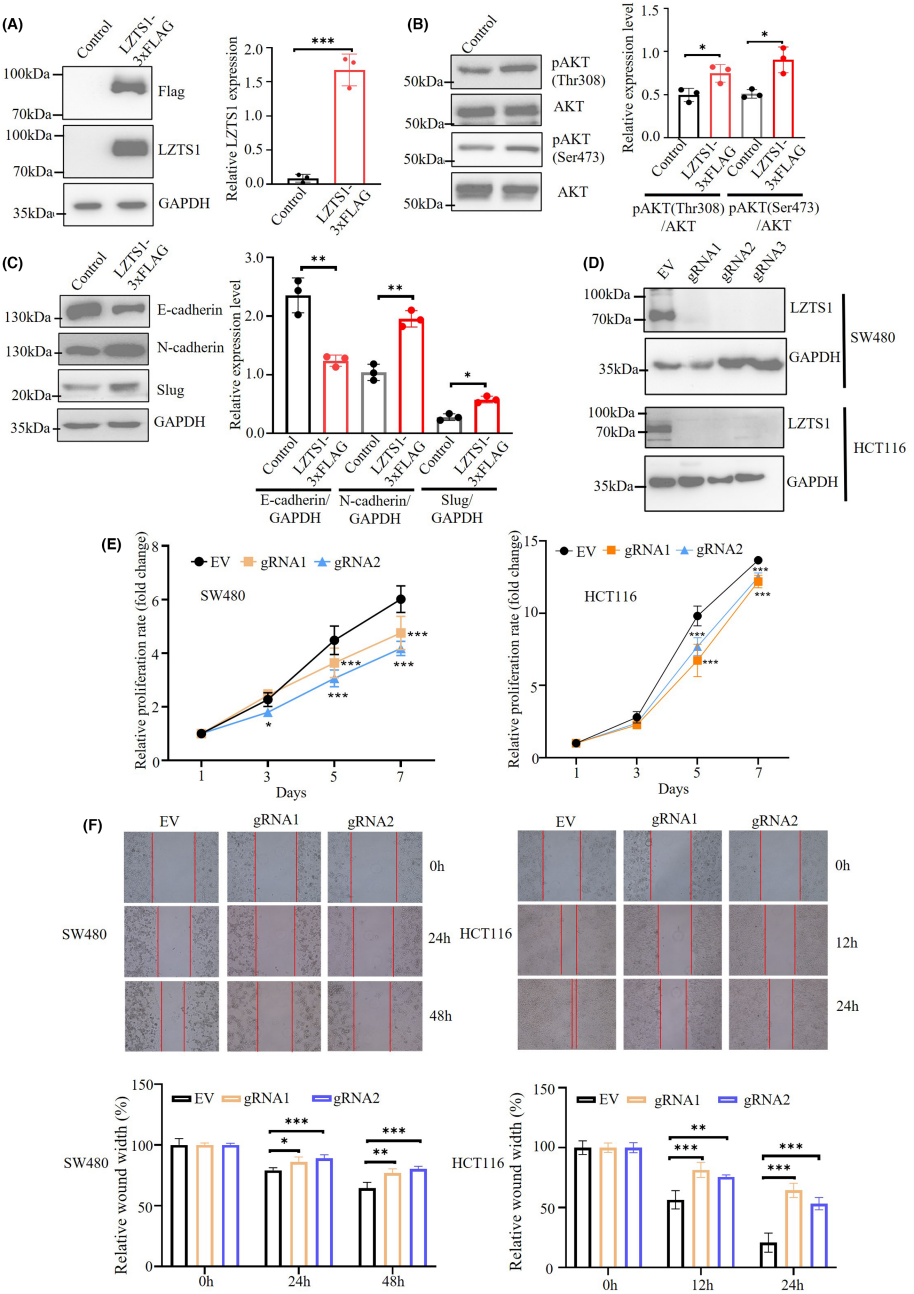
LZTS1 promotes activity of AKT and EMT process and regulates malignant phenotypes of CRC cells. (A) Overexpression of LZTS1 in H293T cells. (B) Overexpression of LZTS1 enhances the activity of AKT at Thr308 and Ser473 site. (C) Overexpression of LZTS1 causes the downregulation of E‐cadherin (the epithelial marker) and upregulation of N‐cadherin and Slug (the mechachymal marker). (D) Protein immunoblotting of cell extracts of SW480 and HCT116 cells transduced by lentivirus with empty vector (EV) or different guide RNAs targeting LZTS1. GAPDH from cell extracts is shown as loading control (lower). (E) Proliferation curves of control cells (EV) and LZTS1 KO cells of SW480 and HCT116. (F) The effect of LZTS1 on the migration rate in SW480 and HCT116 cells. The representative photos of time‐lapse cell migration assay of control cells and LZTS1 KO cells of SW480 and HCT116 cell lines (upper panel), quantitation of migration assays for each CRC cell lines (lower panel). All experiments are performed in triplicate. A two‐tailed Student's *t*‐test was performed for (A). A two‐tailed Student's *t*‐test was performed for (A) a two‐way ANOVA followed by Dunnett's post hoc test was performed for (E) a one‐way ANOVA followed by Dunnett's post hoc test was performed for (F). *p < 0.05, **p < 0.01, ***p < 0.001.

In our pursuit to comprehend the impact of LZTS1 on both the PI3K‐AKT signalling pathway and the epithelial‐mesenchymal transition (EMT) process, we embarked on a series of experiments. Initially, we crafted a LZTS1‐3xFLAG plasmid and successfully introduced it into human cells via transfection. To mitigate potential data noise arising from the inherent cellular heterogeneity characteristic of tumour cell lines, we judiciously selected H293T cells as our preferred experimental model for subsequent investigations. As illustrated in Figure 6A, our efforts resulted in the robust expression of the FLAG tag in the transfected cells. Importantly, we also observed a stark contrast in LZTS1 expression levels between the transfectants and the control group, with LZTS1 being barely detectable in the latter (Figure 6A). These compelling data firmly support the notion that LZTS1 is conspicuously overexpressed in the H293T cells used in this study.

Next, we tend to examine the effect of LZTS1 overexpression on the above‐mentioned signalling pathways. As the Figure 6B indicates, we observed that LZTS1 overexpression enhanced the phosphorylation level of AKT at Ser473 sites, indicating upregulated activity of PI3K‐AKT signalling pathway. Furthermore, we checked the effect of LZTS1 on the EMT process. LZTS1 overexpression caused the decreased expression of epithelial marker E‐cadherin (Figure 6C). In contrast, overexpressed LZTS1 resulted in the increased expression of N‐cadherin and slug, both of which are mesenchymal marker in the EMT process (Figure 6C). In keeping with bioinformatic analysis in tumour samples, LZTS1 promotes the PI3K‐AKT signalling pathway and EMT process, thus contributing to oncogenesis.

### 2.7 LZTS1 inactivation represses the tumorigenic properties of CRC cells

To explore the role of LZTS1 in CRC cells, we established the LZTS1 deficient cell line in SW480 and HCT116 cells using CRISPR‐Cas9 technology. For both SW480 and HCT116 cells, we employed three guide RNAs to inactive LZTS1. As immunoblotting assay showed, LZTS1 expression were barely detected in CRC cells transduced by lentivirus with three guide RNAs (Figure 6D). We subsequently examined whether LZTS1 inactivation could affect the proliferation rates of SW480 and HCT116 cells. We found that LZTS1 inactivation resulted in inhibited proliferation rate in both SW480 and HCT116 cells (Figure 6E). Interestingly, we also found that HCT116 control cells proliferated faster than SW480 control cells (fourteen‐fold vs. six‐fold increase after 7 days, respectively) (Figure 6E), indicating that LZTS1 inactivation represses the proliferation of CRC cells.

We next analysed how LZTS1 impacted on migration ability of CRC cell lines through wound healing assay (Figure 6F). In all HCT116 groups, the wound gap width showed clear decrease trend over time. At 12 h, the wound gap width was decreased by about 45% in control group, while wound gap width of gRNA1 and gRNA2 groups was decreased by 20% and 25%, respectively (Figure 6F), both of which were significantly slower than control group. After 24 h, we also observed that the migration rate of gRNA1 and gRNA2 groups was significantly slower than control group. In contrast, SW480 cells migrated much slower than HCT116 (Figure 6E). Furthermore, we found that the migration rate of gRNA1 and gRNA2 groups was significantly slower than control group in both 24 and 48 h, indicating that disruption of LZTS1 supresses migration rate in CRC (Figure 6F).

### 2.8 The correlation of LZTS1 with PI3K‐AKT pathway and EMT in human CRC tissue

**FIGURE 7:**
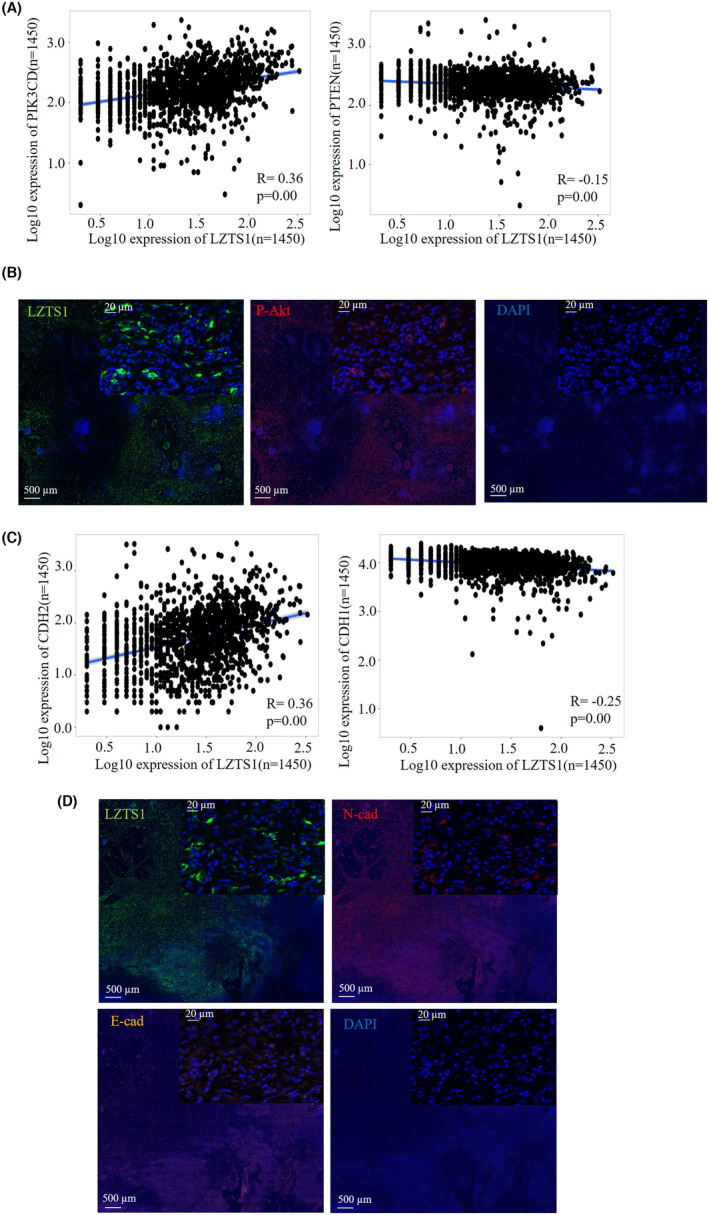
Correlation analysis of LZTS1 with PI3K‐AKT and EMT pathways in CRC. (A) Correlation analysis between mRNA expression of LZTS1 and mRNA expression of the PIK3CD and PTEN in CRC by online TNM plot database. Each dot represents one sample. The strength of the correlation was determined using the Spearman's correlation coefficient (r), and the *p* value was calculated. A linear regression‐fitting curve is shown as a blue line. (B) Tumour tissue from CRC were evaluated by immunofluorescence (IF) for LZTS1 (green signal) and a marker for PI3K‐AKT pathway (pAKT: red signal). (C) Correlation analysis between mRNA expression of LZTS1 and mRNA expression of the CDH1 (E‐cadherin) and CDH2 (N‐cadherin) in CRC by online TNM plot database. Each dot represents one sample. The strength of the correlation was determined using the Spearman's correlation coefficient (R), and the *p* value was calculated. A linear regression‐fitting curve is shown as a blue line (D). (B) Tumour tissue from CRC were evaluated by immunofluorescence (IF) for LZTS1 (Green signal) and the markers for EMT process (N‐cadherin: red signal and E‐cadherin: yellow signal).

To confirm the correlation between the expression of LZTS1 and PI3K‐AKT and EMT pathways, we analysed the mRNA expression of LZTS1, PI3K‐AKT and EMT markers in CRC by TNM plot database. It is well documented that PIK3CD and PTEN are positive and negative regulator for PI3K‐AKT pathway, respectively.^17^ We observed that mRNA expression of LZTS1 positively correlated with mRNA expression of PIK3CD (Figure.7A). In contrast, mRNA expression of LZTS1 negatively correlated with mRNA expression of PTEN (Figure.7A). Immunofluorescence analysis also showed that both LZTS1 and pAKT were consistently high expressed in CRC tumour samples (Figure.7B). These data further support the notion that LZTS1 promotes PI3K‐AKT pathway.

Similarly, gene–gene correlation analysis displayed that the expression of LZTS1 negatively correlated with the expression of CDH1 (encoding E‐cadherin) at mRNA level while its expression positively correlated with the expression of CDH2 (encoding N‐cadherin) (Figure.7C). At protein level, both LZTS1 and N‐cadherin were consistently expressed. In contrast, the expression of LZTS1 and E‐cadherin had a opposite trend (Figure.7D). Taken together, LZTS1 contributes to N‐cadherin expression, thereby promoting EMT process (Figure.7D).

## 3 DISCUSSION

The LZTS1 gene, encoding a 67‐kDa leucin zipper protein, shares a 32% sequence with the cAMP‐responsive activating‐transcription factor (ATF5), suggesting its potential function in regulating DNA transcription.^18^ Further functional analysis showed that LZTS1 can form a complex with phosphatase CDC25C, thus preventing CDC25C from proteasomal degradation during mitosis.^19^ Therefore, loss of LZTS1 causes increased CDC25C degradation during the metaphase, which decreases CDK1 activity. As a result, the decreased CDK1 activity accelerated mitotic progression and improper chromosome segregation.^12^ Furthermore, LZTS1 can regulate microtubule assembly by interacting with p34^cdc2^ at the late S–G2/M stage, and thus impacting mitosis progression.^20^ During the process of neuronal delamination, loss of LZTS1 impairs neuronal migration by reducing the activity of myosin II, indicating that LZTS1 might promote cell migration by regulating microtubule components.^21^ More importantly, this study demonstrated that LZTS1 expression is closely associated with N‐cadherin, an EMT transcriptional factor. Consistently, our data showed that LZTS1 promotes the expression of N‐cadherin, which supports the idea that LZTS1 has a potential role in the EMT process. Recently, Williams et al. classified LZTS1 as a potential epigenetic regulator.^22^ Therefore, further studies are needed to unravel the epigenetic role of LZTS1 in cells.

Since LZTS1 is located at chromosome 8p22, a region of frequent loss of heterozygosity (LOH) in human cancers like breast and bladder cancers,^23^, ^24^ LZTS1 is widely considered a tumour suppressor. However, this notion is recently been challenged by other studies. It has been reported that the expression of LZTS1 is not decreased in ovarian cancer cells, compared to normal ovarian surface epithelial cells, indicating that LZTS1 is not the target of LOH at 8p22 in ovarian cancer.^25^ Furthermore, Welsh et al. reported that the expression of LZTS1 is not altered in primary ovarian tumours than in normal ovary tissues.^26^ Intriguingly, decreased expression of LZTS1 reflects a higher rate of complete response to platinum–taxane‐based chemotherapy than non‐taxane‐based treatment in patients with ovarian cancer,^27^ indicating that a decreased LZTS1 level favours chemotherapy response in ovarian cancer. Moreover, several studies reported that expression of LZTS1 is higher in cancer tissues, compared to matched control samples, indicating the oncogenic function of LZTS1 in carcinogenesis.^28^, ^29^, ^30^, ^31^ Together, these results challenge the notion that LZTS1 functions as a tumour suppressor in cancer. Consistent with these observations, our analysis showed that tumour samples harbour LZTS1 overexpression in multiple cancer types. Interestingly, the increment of LZTS1 expression is more in endometrial cancer patients without lymph node metastasis, compared to endometrial cancer patients with lymph node metastasis.^31^ Furthermore, Simoes et al. reported that LZTS1 expression is strongly associated with the development of metastasis,^32^ indicating that LZTS1 promotes cancer metastasis.

Recently, Hong et al. demonstrated that LZTS1 is overexpressed in the intestinal mucosa of Crohn's disease (CD) patients, compared to the health control group.^33^ More importantly, LZTS1 expression is much higher in inflamed intestinal mucosa of CD patients than that of noninflamed intestinal mucosa of CD patients, suggesting that high expression of LZTS1 promotes inflammatory symptoms in CD, thus increasing the CRC risk. Accordingly, our study found that high expression of LZTS1 is associated with poor prognosis in CRC patients. In keeping with these findings, Ma et al. reported that expression of LZTS1 is negatively associated with OS in CRC patients.^28^

The PI3K‐Akt signalling pathway, a classical oncogenic pathway, has been widely reported to promote carcinogenesis.^34^ In our study, we found that many upregulated DEGs are enriched in this pathway in the high LZTS1^high^ CRC patient' group, indicating that high expression of LZTS1 contributes to the activity of PI3K‐AKT signalling pathway. Consistently, exogenous expression of LZTS1 in H293T cells upregulate phosphorylated AKT at both the Thr308 and Ser473 sites. In keeping with this finding, one study reported that overexpression of LZTS1 upregulates the activity of the AKT/GSK‐3β signalling pathway in pancreatic cancer cells.^15^ On the other hand, another study reported that LZTS1 could be a negative regulator of AKT, thus inhibiting carcinogenesis.^35^ Consequently, a comprehensive understanding of the precise mechanisms governing LZTS1's impact on the PI3K‐AKT pathway holds potential significance for tailoring therapeutic strategies in cancer treatment.

In this study, we showed that the expression of LZTS1 was higher in CRC tissues as well as multiple other cancer types compared to normal tissues, and its overexpression was correlated with poor survival in CRC. In CRC tissues, LZTS1 overexpression was significantly correlated to several clinical indices linked to tumour progression like tumour grade and lymph node status. Accordingly, the LZTS1 overexpression in the CRC tissues was associated with lower methylation of the LZTS1 promoter, in contrast to normal colorectal tissues. Furthermore, molecular function analysis revealed that high expression of LZTS1 in CRC tissues largely contributes to PI3K‐AKT and EMT biological processes. Our experimental data further supports that LZTS1 overexpression upregulated phosphorylated AKT and mesenchymal markers like N‐cadherin and slug. Furthermore, inhibition of LZTS1 could repress the tumorigenic properties of CRC cells. These data further support the idea that LZTS1 could play an oncogenic function in colorectal carcinogenesis.

## 4 METHODS AND MATERIALS

### 4.1 Data acquisition

All the bioinformatic analyses were performed by the R software. The package TCGAbiolinks was used to download the RNA‐seq data for the COAD‐READ tumour types. In total there are 698 samples distributed as follows: primary solid tumour (647) and solid normal tissue (51). Before running the differential analysis with the R‐package LIMMA,^36^ a pre‐filtering step was applied. In this way, we removed low expressed genes by the LIMMA filterByExpr function. In the next step, the data were normalized using the Voom method.^37^ Patients without clinical data information were excluded from the related clinical prognosis analysis. Two expression microarray series, that is GSE41258 and GSE87211 containing CRC tumour and paired normal tissues, were retrieved from the Gene Expression Omnibus (GEO, https://www.ncbi.nlm.nih.gov/geo/). The details of each GEO microarray series are summarized in Table S2. TIMER was used to analyse the expression of LZTS1 across various tumour (https://cistrome.shinyapps.io/timer/).^38^

### 4.2 KEGG and GO analysis

In the TCGA dataset, we acquired these DEGs between LZTS1^low^ and LZTS1^high^ groups identified based on the median cut‐off of LZTS1 expression using the ‘edgeR’ package. Fold change >1.5 and adjusted *p* value <0.05 were used as the standards for DEGs. To determine the functions of the 1635 upregulated DEGs and 1622 upregulated DEGs, these DEGs were then subjected to DAVID 6.8 (https://david.ncifcrf.gov/) for KEGG pathway and GO functional analyses. The results of downregulated DEGs were shown in Figure S1A–D.

### 4.3 GSEA analysis

To explore the expressive differences between LZTS1^low^ and LZTS1^high^ subgroups and diverse pathways within them, the R package FGSEA^39^ was used to test the significance of a biological pathway. With the R package clusterProfiler^40^ we performed an enrichment analysis on the significant genes. The gene set permutations were performed 1000 times to obtain the normalized enrichment score.

### 4.4 Survival analysis

Firstly, Cox regression analysis was performed to estimate the relationship between LZTS1 expression and OS, DSS and progression‐free survival (PFS) in CRC from the TCGA database using the Sangerbox website (http://sangerbox.com).^41^

### 4.5 Expression of LZTS1 in different CRC subtypes and DNA methylation analysis

The UALCAN database was used to compare the expression of LZTS1 in different CRC subtypes based on the TCGA dataset.^42^ In addition, DNA methylation level of the LZTS1 promoter in tumours and corresponding normal tissues was analysed by the UALCAN database.

### 4.6 The correlation analysis of gene expression in CRC

The TNMplot database^43^ was used to analyse the correlation between the expression of LZTS1 and expression of AKT, E‐cadherin, N‐cadherin and Slug in different CRC subtypes based on the TCGA dataset and GEO datasets.

### 4.7 Clinical samples

The study was conducted in accordance with the Declaration of Helsinki. The tumour and adjacent nontumor tissues used in this study were obtained via written informed consent from the patients with CRC undergoing colon cancer resection in the Department of General Surgery of Henan Tongxu County People' Hospital. The experimental protocols for immunohistochemistry and tissue microarray were approved by the Ethics Committee of Henan Tongxu County People's Hospital, which granted research ethics approval for this study. All human samples for immunofluorescence were obtained following ethical approval from the Commissie Medische Ethiek UZ KU Leuven/Onderzoek (Ethics no.: S66460) and individuals signed an informed consent.

### 4.8 Tissue microarrays

Tissue microarrays were constructed by Shanghai Wellbio Technology Co., Ltd (Wellbio Technology Co., Shanghai, China). Pathologists‐stained tissue paraffin blocks of CRC paired samples from test and validation cohorts with haematoxylin–eosin to confirm the diagnoses and marked at fixed points which displayed the most typical histological characteristics under a microscope. Cores with 1.0‐mm diameter from per‐donor block were diverted into a recipient block microarrayer, and each dot array contained fewer than 180 dots. 4‐μm‐thick sections were cut from the recipient block and diverted to glass slides used with an adhesive tape transfer system in order to immunohistochemistry.

### 4.9 Immunohistochemistry

Specimens were paraffin‐embedded. Serial 4 μm sections were cut, deparaffinized, blocked and incubated at 4°C overnight with the primary antibody, followed by a horseradish peroxidase‐labelled secondary antibody. The primary antibody used is LZTS1 polyclonal antibody purchased from ThermoFisher. Human CRC tissue microarrays (ZL‐CocSur 1801) were purchased from Shanghai Zhuolibiotech Company Co., Ltd. (Shanghai, China), and LZTS1 expression was evaluated using the automated VIS DIA VisioMorph System (Visiopharm®, Hoersholm, Denmark). Clinical and pathological information concerning the samples is summarized in Table S3.

### 4.10 Immunofluorescence assay

Formalin‐fixed, paraffin‐embedded (FFPE) material was obtained from surgical resection specimens at University Hospital Leuven (UZ Leuven), in the form of 4–6 μm‐sections of one tissue block per patient. Antigen retrieval was conducted on deparaffinized sections using citrate buffer (pH 6.0) in a microwave oven. Subsequently, the slides were washed in TBS containing 0.01% Tween. Sections were treated with ENZO Peroxidase block for 15 min to eliminate endogenous peroxidase activity, followed by a 2‐min wash in TBS‐Tween. Then, sections were incubated with a protein block solution comprising 5% BSA, 2% milk, and 1% human IgG in TBS‐Tween for 1 h. Following this, sections were exposed to anti‐LZTS1 polyclonal antibody (Thermofisher) for 1 h at room temperature. Negative controls were prepared by omitting the primary antibody. Slides were rinsed three times in TBS‐Tween and further incubated for 1 h with Polymer‐HRP goat anti‐rabbit (DAKO) diluted 1:200, followed by three washes in TBS‐Tween. Next, slides were incubated in Tyramide Signal Amplification in borate buffer (1 M borate, 3 M NaCl, 0.1% Tween adjusted to pH 7.8 containing 0.003% H_2_O_2_) for 10 min. They were then rinsed three times with TBS‐Tween and incubated with Hoesch for nuclei staining (Cell Signalling Technology). The same procedure was subsequently performed for anti‐E‐cadherin monoclonal (Cell Signalling Technology), anti‐Phospho‐Akt (Ser473) (Cell Signalling Technology) rabbit mAb (4 Cell Signalling Technology), and anti‐N‐cadherin antibody clone 13A9 (Cell Signalling Technology).

### 4.11 Cell culture and transfection

HEK‐293 T and the human CRC cell lines SW480 and HCT116 were obtained from the American Type Culture Collection. HEK‐293 T cells and SW480 cells were cultured in DMEM supplemented with 10% fetal bovine serum and1% penicillin–streptomycin (Invitrogen). Human CRC cell line HCT116 was maintained in McCoy's 5A (Modified) medium supplemented with 10% fetal bovine serum and 1% penicillin–streptomycin (Life Technologies). The cell lines were routinely tested for mycoplasma and maintained at 37°C in a humidified atmosphere with 5% CO_2_. For transfection, LZTS1 with 3xFlag label overexpressing plasmid was purchased from Guangzhou GeneCopoeia, Inc. (Guangzhou, China). For transfection, 2ug LZTS1‐3xFlag plasmid was added to serum‐free DMEM medium and then 4 μL (1 mg/mL) PEI was added to the diluted plasmid. This mixture was incubated 15–20 min at room temperature. Next, the transfection mix was transferred to the H293T cells. After 18 h, H293T cells were harvested for later use.

### 4.12 Immunoblotting

Cells were lysed in RIPA lysis buffer with protease inhibitor and phosphatase inhibitors cocktail (Cell Signalling Technology). After brief sonication, the preparation was centrifuged, and the supernatants were qualified using a Pierce™ BCA protein assays kit (Thermo Fisher Scientific) following the manufacturer's protocol. Proteins were resolved on 8% SDS‐PAGE gels and transferred to a nitrocellulose membrane. After blocking with 5% non‐fat milk in PBS with 0.1% Tween‐20, membranes were incubated with the primary antibody followed by the secondary antibody conjugated with horseradish peroxidase. After washing, the bands were visualized with enhanced chemiluminescence substrate and quantified using Image J software. Detailed information on the antibodies used is given in the supplementary data (Table S4).

### 4.13 CRISPR‐Cas9‐based LZTS1 knockout

sgRNAs (oligonucleotide sequences were indicated in Table S5) were ligated into BsmBI‐digested lentiCRISPR‐v2 with T4 ligase (NEB). In order to produce lentivirus, HEK‐293 T cells were resuspended in DMEM medium and co‐transfected with 8 μg of gRNA expression or lentiCRISPR‐v2 (EV) constructs, 4 μg psPAX2 vector (Addgene), and 2 μg psMD2G (Addgene) vector in 28 μL of PEI. The medium was aspirated after 16 h and replaced with fresh DMEM/F12 with 10% FCS. The supernatant was collected after 48 h, centrifuged at 1500 rpm at 4°C for 5 min, filtered through a 0.45 μm low protein‐binding membrane (Millipore), and used to transduce CRC cells in the presence of 8 mg/mL polybrene (Sigma). The virus‐containing media were removed 24 h after transduction, and infected cells were screened with puromycin (1.5 μg/mL). The expression of LZTS1 was evaluated by an immunoblotting assay.

### 4.14 Cell proliferation assay

Cell viability was examined using a Cell Counting Kit‐8 (CCK8) cell proliferation assay according to the manufacturer's protocol. Briefly, cells were counted and plated at a density of 1 × 10^3^ cells per well in 96‐well plates in nonuple and cultured at 37°C with 5% CO_2_ in a humidified incubator. CCK8 reagent was added at 1, 3, 5, or 7 days, and incubation was continued for an additional 2 h. A colorimetric assay was done using a microplate reader at a wavelength of 450 nm (ThermoFisher Scientific, Varioskan™ LUX).

### 4.15 Cell migration assay

Cells were seeded at a density of 3 × 10^4^ cells per well in an attached silicone insert with a defined cell‐ free gap (Ibidi) and cultured for 12–24 h to reach 80% of confluence. The wound was generated by removing inserts. Medium was changed to remove dead cells and. The images were taken by a light microscope (×10 magnification) fitted with a microscope camera (Leica) at time intervals of 0, 12 and 24 h. Cell migration towards the wound was calculated as a percentage of wound closure: percentage of wound closure = DT/D0 × 100%, where D0 is the area of wound gap measured immediately after the wound gap was made, and DT is the area of wound measured 12 or 24 h after the wound gap was made.

### 4.16 Statistical analysis

Quantitative data were analysed using Student's *t*‐test. Survival curves were generated by the Kaplan–Meier method. The log‐rank test was used to determine the significance of differences between survival curves. For three or more groups, the statistical significance was analysed using one‐way by Dunnett's post hoc test. Statistical analysis was performed using Prism (GraphPad). A value of *p* < 0.05 was regarded as indicative of statistical significance. Data are provided as means ± standard deviations (SDs).

## AUTHOR CONTRIBUTIONS

**Yuanchun Xu:** Conceptualization (lead); investigation (equal); writing – original draft (equal). **Daniele Pepe:** Conceptualization (equal); investigation (equal); methodology (equal); resources (equal). **Shu Yao:** Investigation (supporting); methodology (equal). **Loubna Boudhan:** Investigation (equal). **Sara Verbandt:** Investigation (equal). **Ting Pu:** Investigation (equal). **John W. M. Creemers:** Validation (equal); writing – review and editing (equal). **Maoxuan Liu:** Data curation (equal); formal analysis (equal). **Sabine Tejpar:** Resources (equal). **Zongsheng He:** Conceptualization (equal); funding acquisition (equal); investigation (equal); methodology (equal); writing – original draft (equal). **Jingjing Zhu:** Conceptualization (equal); funding acquisition (equal); investigation (equal); methodology (equal); supervision (equal); writing – review and editing (equal). **Yaling Wang:** Supervision (equal); writing – review and editing (equal).

## FUNDING INFORMATION

Y.X. received grants from Nursing innovative project of Army medical center (grant number: 2023HLCXZ04). Z.H. received grants from the National Natural Science Foundation of China (grant number: 82203318) and Natural Science Foundation of Chongqing (grant number: CSTB2022NSCQ‐MSX0880). J.Z. was supported by Fondation Contre le Cancer (grant number: 2019‐094) and WELBIO (Walloon Excellence in Life Sciences and Biotechnology, grant number: WELBIO‐CR‐2019C‐05).

## CONFLICT OF INTEREST STATEMENT

The authors declare that they have no competing interests.

## Supporting information


**Figure S1.** The KEGG and GO analysis of 1622 downregulated DEGs. (A) Pathways enrichment of 1622 downregulated DEGs by KEGG analysis. (B–D). Biological process, molecular function and cellular component of 1622 downregulated DEGs by GO analysis.

**Table S1.** The DEGs based on the LZTS1 expression in TCGA CRC samples.

**Table S2.** Expression level of LZTS1 in different groups from GSE41258 and GSE87211 datasets.

**Table S3.** Clinical and pathological information of CRC tumour tissue microarray.

**Table S4.** Antibodies used in this study.

**Table S5.** Oligonucleotides used in this study.

## Data Availability

RNA‐seq and clinical data of patients were retrieved from the TCGA database (https://gdc.cancer.gov). The microarray datasets (GSE41258 and GSE87211) were downloaded from GEO (https://www.ncbi.nlm.nih.gov/geo/). The R package and computer code are available from the corresponding author upon request.

## References

[1] Siegel RL , Wagle NS , Cercek A , Smith RA , Jemal A . Colorectal cancer statistics, 2023. CA Cancer J Clin. 2023;73:233‐254. 36856579 10.3322/caac.21772

[2] Xi Y , Xu P . Global colorectal cancer burden in 2020 and projections to 2040. Transl Oncol. 2021;14:101174. 34243011 10.1016/j.tranon.2021.101174 PMC8273208

[3] Xu H , Liu L , Li W , et al. Transcription factors in colorectal cancer: molecular mechanism and therapeutic implications. Oncogene. 2021;40:1555‐1569. 33323976 10.1038/s41388-020-01587-3

[4] Nakayama M , Oshima M . Mutant p53 in colon cancer. J Mol Cell Biol. 2019;11:267‐276. 30496442 10.1093/jmcb/mjy075 PMC6487790

[5] Jung G , Hernández‐Illán E , Moreira L , Balaguer F , Goel A . Epigenetics of colorectal cancer: biomarker and therapeutic potential. Nat Rev Gastroenterol Hepatol. 2020;17:111‐130. 31900466 10.1038/s41575-019-0230-y PMC7228650

[6] Itatani Y , Kawada K , Sakai Y . Transforming growth factor‐β signaling pathway in colorectal cancer and its tumor microenvironment. Int J Mol Sci. 2019;20:5822. 31756952 10.3390/ijms20235822 PMC6929101

[7] Soleimani A , Pashirzad M , Avan A , Ferns GA , Khazaei M , Hassanian SM . Role of the transforming growth factor‐β signaling pathway in the pathogenesis of colorectal cancer. J Cell Biochem. 2019;120:8899‐8907. 30556274 10.1002/jcb.28331

[8] He Z , Khatib A‐M , Creemers JWM . The proprotein convertase furin in cancer: more than an oncogene. Oncogene. 2022;41:1252‐1262. 34997216 10.1038/s41388-021-02175-9

[9] He Z , Thorrez L , Siegfried G , et al. The proprotein convertase furin is a pro‐oncogenic driver in KRAS and BRAF driven colorectal cancer. Oncogene. 2020;39:3571‐3587. 32139876 10.1038/s41388-020-1238-z

[10] Chen L , Zhu Z , Sun X , et al. Down‐regulation of tumor suppressor gene FEZ1/LZTS1 in breast carcinoma involves promoter methylation and associates with metastasis. Breast Cancer Res Treat. 2009;116:471‐478. 18686028 10.1007/s10549-008-0147-6 PMC2927198

[11] Nonaka D , Fabbri A , Roz L , et al. Reduced FEZ1/LZTS1 expression and outcome prediction in lung cancer. Cancer Res. 2005;65:1207‐1212. 15735004 10.1158/0008-5472.CAN-04-3461

[12] Vecchione A , Baldassarre G , Ishii H , et al. Fez1/Lzts1 absence impairs Cdk1/Cdc25C interaction during mitosis and predisposes mice to cancer development. Cancer Cell. 2007;11:275‐289. 17349584 10.1016/j.ccr.2007.01.014 PMC1987708

[13] Zhou W , He MR , Jiao HL , et al. The tumor‐suppressor gene LZTS1 suppresses colorectal cancer proliferation through inhibition of the AKT‐mTOR signaling pathway. Cancer Lett. 2015;360:68‐75. 25667121 10.1016/j.canlet.2015.02.004

[14] He Y , Liu X . The tumor‐suppressor gene LZTS1 suppresses hepatocellular carcinoma proliferation by impairing PI3K/Akt pathway. Biomed Pharmacother. 2015;76:141‐146. 26653561 10.1016/j.biopha.2015.10.006

[15] Liu L , Chen L , Yang J , et al. LZTS1 promotes proliferation and suppresses apoptosis by inhibiting the activation of AKT/GSK‐3β signaling pathway in pancreatic cancer cells. Trop J Pharm Res. 2022;21:31‐36.

[16] Dhar GA , Saha S , Mitra P , Nag Chaudhuri R . DNA methylation and regulation of gene expression: guardian of our health. Nucleus. 2021;64:259‐270. 10.1007/s13237-021-00367-y PMC8366481 34421129

[17] Vidotto T , Melo CM , Castelli E , Koti M , dos Reis RB , Squire JA . Emerging role of PTEN loss in evasion of the immune response to tumours. Br J Cancer. 2020;122:1732‐1743. 32327707 10.1038/s41416-020-0834-6 PMC7283470

[18] Cabeza‐Arvelaiz Y , Sepulveda JL , Lebovitz RM , Thompson TC , Chinault AC . Functional identification of LZTS1 as a candidate prostate tumor suppressor gene on human chromosome 8p22. Oncogene. 2001;20:4169‐4179. 11464283 10.1038/sj.onc.1204539

[19] Baldassarre G , Croce CM , Vecchione A . Take your “M” time. Cell Cycle. 2007;6:2087‐2090. 17873519 10.4161/cc.6.17.4628

[20] Ishii H , Vecchione A , Murakumo Y , et al. FEZ1/LZTS1 gene at 8p22 suppresses cancer cell growth and regulates mitosis. Proc Natl Acad Sci USA. 2001;98:10374‐10379. 11504921 10.1073/pnas.181222898 PMC56968

[21] Kawaue T , Shitamukai A , Nagasaka A , et al. Lzts1 controls both neuronal delamination and outer radial glial‐like cell generation during mammalian cerebral development. Nat Commun. 2019;10:1‐18. 31239441 10.1038/s41467-019-10730-y PMC6592889

[22] Williams RT , Guarecuco R , Gates LA , et al. ZBTB1 regulates asparagine synthesis and leukemia cell response to L‐asparaginase. Cell Metab. 2020;31:852‐861. e6. 32268116 10.1016/j.cmet.2020.03.008 PMC7219601

[23] Stoehr R , Wissmann C , Suzuki H , et al. Deletions of chromosome 8p and loss of sFRP1 expression are progression markers of papillary bladder cancer. Lab Investig. 2004;84:465‐478. 14968126 10.1038/labinvest.3700068

[24] Anbazhagan R , Fujii H , Gabrielson E . Allelic loss of chromosomal arm 8p in breast cancer progression. Am J Pathol. 1998;152:815‐819. 9502423 PMC1858406

[25] Arnold JM , Choong DYH , Lai J , Campbell IG , Chenevix‐Trench G . Mutation and expression analysis of LZTS1 in ovarian cancer. Cancer Lett. 2006;233:151‐157. 15876481 10.1016/j.canlet.2005.03.008

[26] Welsh JB , Zarrinkar PP , Sapinoso LM , et al. Analysis of gene expression profiles in normal and neoplastic ovarian tissue samples identifies candidate molecular markers of epithelial ovarian cancer. Proc Natl Acad Sci USA. 2001;98:1176‐1181. 11158614 10.1073/pnas.98.3.1176 PMC14728

[27] Califano D , Pignata S , Pisano C , et al. FEZ1/LZTS1 protein expression in ovarian cancer. J Cell Physiol. 2010;222:382‐386. 19885841 10.1002/jcp.21962

[28] Ma J , Wang P , Huang L , Qiao J , Li J . Bioinformatic analysis reveals an exosomal miRNA‐mRNA network in colorectal cancer. BMC Med Genet. 2021;14:1‐18. 10.1186/s12920-021-00905-2 PMC7913431 33639954

[29] Zhang L , Xiao H , Zhou H , et al. Development of transcriptomic biomarker signature in human saliva to detect lung cancer. Cell Mol Life Sci. 2012;69:3341‐3350. 22689099 10.1007/s00018-012-1027-0 PMC4121486

[30] Bai KH , He SY , Shu LL , et al. Identification of cancer stem cell characteristics in liver hepatocellular carcinoma by WGCNA analysis of transcriptome stemness index. Cancer Med. 2020;9:4290‐4298. 32311840 10.1002/cam4.3047 PMC7300398

[31] López‐Ozuna VM , Kogan L , Hachim MY , et al. Identification of predictive biomarkers for lymph node involvement in obese women with endometrial cancer. Front Oncol. 2021;11:1‐11. 10.3389/fonc.2021.695404 PMC8292832 34307159

[32] Simões CC , Call MK , Corrêa ZM , Spaulding AG , Augsburger JJ . Clinical and histopathological features and immunoreactivity of human choroidal and ciliary melanomas as prognostic factors for metastasis and death. Graefes Arch Clin Exp Ophthalmol. 2011;249:1795‐1803. 21847577 10.1007/s00417-011-1769-7

[33] Hong SN , Joung JG , Bae JS , et al. RNA‐seq reveals transcriptomic differences in inflamed and noninflamed intestinal mucosa of Crohn's disease patients compared with normal mucosa of healthy controls. Inflamm Bowel Dis. 2017;23:1098‐1108. 28613228 10.1097/MIB.0000000000001066

[34] Noorolyai S , Shajari N , Baghbani E , Sadreddini S , Baradaran B . The relation between PI3K/AKT signalling pathway and cancer. Gene. 2019;698:120‐128. 30849534 10.1016/j.gene.2019.02.076

[35] Guanen Q , Junjie S , Baolin W , et al. MiR‐214 promotes cell meastasis and inhibites apoptosis of esophageal squamous cell carcinoma via PI3K/AKT/mTOR signaling pathway. Biomed Pharmacother. 2018;105:350‐361. 29864623 10.1016/j.biopha.2018.05.149

[36] Ritchie ME , Phipson B , Wu D , et al. Limma powers differential expression analyses for RNA‐sequencing and microarray studies. Nucleic Acids Res. 2015;43:e47. 25605792 10.1093/nar/gkv007 PMC4402510

[37] Law CW , Chen Y , Shi W , Smyth GK . Voom: precision weights unlock linear model analysis tools for RNA‐seq read counts. Genome Biol. 2014;15:1‐17. 10.1186/gb-2014-15-2-r29 PMC4053721 24485249

[38] Li T , Fu J , Zeng Z , et al. TIMER2.0 for analysis of tumor‐infiltrating immune cells. Nucleic Acids Res. 2020;48:W509‐W514. 32442275 10.1093/nar/gkaa407 PMC7319575

[39] Korotkevich G , Sukhov V , Budin N , et al. Fast Gene Set Enrichment Analysis. bioRXiV. 2016. doi:10.1101/060012

[40] Wu T , Hu E , Xu S , et al. clusterProfiler 4.0: a universal enrichment tool for interpreting omics data. Innovations. 2021;2:100141. 10.1016/j.xinn.2021.100141 PMC8454663 34557778

[41] Shen W , Song Z , Zhong X , et al. Sangerbox: a comprehensive, interaction‐friendly clinical bioinformatics analysis platform. iMeta. 2022;1:e36. 38868713 10.1002/imt2.36 PMC10989974

[42] Chandrashekar DS , Karthikeyan SK , Korla PK , et al. UALCAN: an update to the integrated cancer data analysis platform. Neoplasia. 2022;25:18‐27. 35078134 10.1016/j.neo.2022.01.001 PMC8788199

[43] Bartha Á , Győrffy B . TNMplot.Com: a web tool for the comparison of gene expression in normal, tumor and metastatic tissues. Int J Mol Sci. 2021;22:1‐12. 10.3390/ijms22052622 PMC7961455 33807717

