# Supplementary figures and images for: Unveiling the oncogenic role of LZTS1 in colorectal cancer
Source: J Cell Mol Med. 2024 Jul 18;28(14):e18441. doi: 10.1111/jcmm.18441 (PMC11256987; doi:10.1111/jcmm.18441)

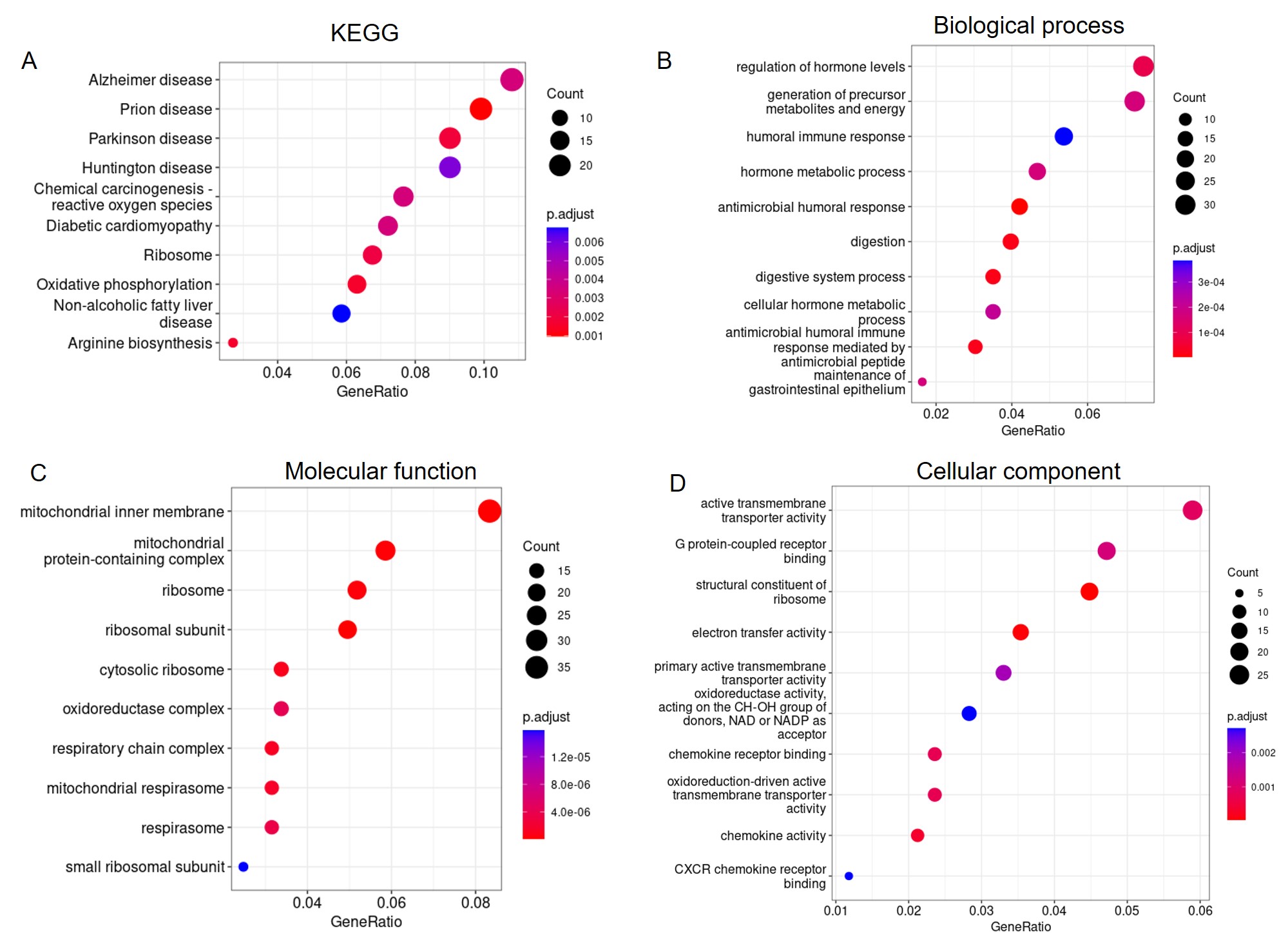

Supplement: Supplementary file 1 — Figure S1. The KEGG and GO analysis of 1622 downregulated DEGs. (A) Pathways enrichment of 1622 downregulated DEGs by KEGG analysis. (B–D). Biological process, molecular function and cellular component of 1622 downregulated DEGs by GO analysis. [file JCMM-28-e18441-s006.jpg]
