# Supplementary material for: Unveiling the oncogenic role of LZTS1 in colorectal cancer
Source: J Cell Mol Med. 2024 Jul 18;28(14):e18441. doi: 10.1111/jcmm.18441 (PMC11256987; doi:10.1111/jcmm.18441)
Supplement: Supplementary file 5 — Table S4. Antibodies used in this study. [file JCMM-28-e18441-s004.docx]

| Antibodies | Source | Identifier |
| --- | --- | --- |
| Mouse monoclonal anti-GAPDH  Goat anti-rabbit HRP-conjugated 2ary antibody  Goat anti-mouse HRP-conjugated 2ary antibody  Rabbit polyclonal anti-human LZTS1  Rabbit polyclonal anti-human LZTS1  Rabbit polyclonal anti-phospho-AKT1-T308  Rabbit monoclonal anti-E-Cadherin(24E10)  Rabbit monoclonal anti-Slug(C19G7)  Rabbit monoclonal anti-N-Cadherin(D4R1H)  Rabbit monoclonal anti-AKT  Rabbit monoclonal anti-phospho-AKT(Ser473)(D9E)  Mouse monoclonal anti-Flag | Proteintech ZSJQ  ZSJQ  Thermofisher  Abclonal  Abclonal  CST  CST  CST  Abclonal  CST  Proteintech | Cat# 60004-1-lg  Cat# ZB-2301  Cat# ZB-2305  Cat#PA5-52274  Cat#A16496  Cat#AP0304  Cat#3195  Cat#9585  Cat#13116  Cat#A18675  Cat#4060  Cat#66008-4-lg |

**Table S4. Antibodies used in this study**
