# Supplementary material for: Unveiling the oncogenic role of LZTS1 in colorectal cancer
Source: J Cell Mol Med. 2024 Jul 18;28(14):e18441. doi: 10.1111/jcmm.18441 (PMC11256987; doi:10.1111/jcmm.18441)
Supplement: Supplementary file 6 — Table S5. Oligonucleotides used in this study. [file JCMM-28-e18441-s001.docx]

| Primer Name | Sequence |
| --- | --- |
| sgLZTS1-1F  sgLZTS1-1R  sgLZTS1-2F  sgLZTS1-2R  sgLZTS1-3F  sgLZTS1-3R | caccgGCTCTGTCCTTCTCCGACGG  aaacCCGTCGGAGAAGGACAGAGC  caccgGGAGCTCGAGAGCCTCATGA  aaacTCATGAGGCTCTCGAGCTCC  caccgGAGGGCCCGGAGCCCAAAGG  aaacCCTTTGGGCTCCGGGCCCTC |

**Table S5. Oligonucleotides used in this study.**
